# Comparison between Field Effect Transistors and Bipolar Junction Transistors as Transducers in Electrochemical Sensors

**DOI:** 10.1038/srep41430

**Published:** 2017-01-30

**Authors:** Sufi Zafar, Minhua Lu, Ashish Jagtiani

**Affiliations:** 1 IBM T.J. Watson Research Center, Yorktown Heights, NY, 10598, USA.

## Abstract

Field effect transistors (FET) have been widely used as transducers in electrochemical sensors for over 40 years. In this report, a FET transducer is compared with the recently proposed bipolar junction transistor (BJT) transducer. Measurements are performed on two chloride electrochemical sensors that are identical in all details except for the transducer device type. Comparative measurements show that the transducer choice significantly impacts the electrochemical sensor characteristics. Signal to noise ratio is 20 to 2 times greater for the BJT sensor. Sensitivity is also enhanced: BJT sensing signal changes by 10 times per pCl, whereas the FET signal changes by 8 or less times. Also, sensor calibration curves are impacted by the transducer choice. Unlike a FET sensor, the calibration curve of the BJT sensor is independent of applied voltages. Hence, a BJT sensor can make quantitative sensing measurements with minimal calibration requirements, an important characteristic for mobile sensing applications. As a demonstration for mobile applications, these BJT sensors are further investigated by measuring chloride levels in artificial human sweat for potential cystic fibrosis diagnostic use. In summary, the BJT device is demonstrated to be a superior transducer in comparison to a FET in an electrochemical sensor.

The application of field effect transistors (FET) as transducers in electrochemical sensors was first described in 1970 by Bergveld^1^. Since then, field effect transistor (FET) based electrochemical sensors^2^,^3^,^4^,^5^,^6^,^7^,^8^,^9^,^10^,^11^ have been extensively investigated due to their enhanced sensitivity, resolution, low power, portability and fabrication compatibility with silicon processing technology. Over the years, these electrochemical sensors have been demonstrated to detect both ions and biomolecules^2^,^3^,^4^,^5^,^6^,^7^,^8^,^9^, and have several sensing applications, including DNA sequencing^12^,^13^ and mobile diagnostics^14^,^15^. Recently, bipolar junction transistor (BJT) device^16^,^17^,^18^ was proposed as a transducer in an electrochemical sensor. In this study, we compare a FET transducer with the recently proposed BJT transducer. The objective is to evaluate the impact of the transducer type on the performance metrics of an electrochemical sensor.

An electrochemical sensor has two basic components as shown in Fig. 1: the sensing surface (or receptor) and the transducer. The sensing surface interacts with the target analyte and the transducer converts this interaction into a readable electronic signal. The sensor performance characteristics depend on both the components. The sensor selectivity and affinity towards the target analyte depends solely on the sensing surface because the analyte interacts only at the sensing surface as illustrated in Fig. 1. Other performance metrics such as sensitivity, resolution, and calibration depend on both components. Since the present objective is to compare the performance of a FET transducer with that of a BJT transducer in an electrochemical sensor, it is important to ensure that the sensing surface does not impact the evaluation. If the sensing surface and target analyte solution are kept the same, the sensing surface selectivity and target analyte affinity would therefore remain the same; in that case the observed differences in the electrochemical sensor performance metrics could then be attributed to transducers. In other words, electrochemical sensors that are identical in all details except for the transducer device type (FET versus BJT) must be used to ensure that the transducer comparison is accurate.

In the present study, electrochemical sensors with FET and BJT as transducers are compared. Both the sensors have the same chloride ion sensitive silver chloride sensing surface. Measurements show that the recently proposed BJT transducer is superior in comparison to the widely used FET transducer: both sensitivity and signal to noise ratio (SNR) are significantly higher for BJT sensors. More importantly, SNR, sensitivity and calibration curves are independent of measurement voltages for the BJT sensor. This implies that optimal measurements can be made over the entire sensing range with minimal calibration requirements which is a significant advantage, particularly for mobile diagnostic applications. In contrast, sensitivity, resolution and calibration curves are dependent on the applied gate voltage for a FET sensor. Consequently, for the FET sensor, detailed calibration curves are required to obtain accurate quantitative sensing results and also use voltages have to be carefully chosen to ensure maximum sensitivity and resolution. As a demonstration for mobile diagnostic applications, the BJT sensor is further investigated for diagnosing cystic fibrosis which requires accurate measurement of chloride ion in sweat^19^,^20^,^21^. Sensing measurements are shown to be repeatable and reversible in artificial human sweat. In summary, the BJT is shown to be a superior transducer option for an electrochemical sensor.

## Results and Discussion

In this section, electrochemical sensors with FET and BJT devices as transducers are compared. These electrochemical sensors are identical except for the transducer device type to ensure that the transducer comparison is accurate. For the comparison purpose, we have chosen chloride ion sensitive silver chloride sensing surface. Also, these electrochemical sensors are further investigated by for potential cystic fibrosis diagnostic applications.

### Electrochemical sensors with FET and BJT as transducers

**Figure 2:**
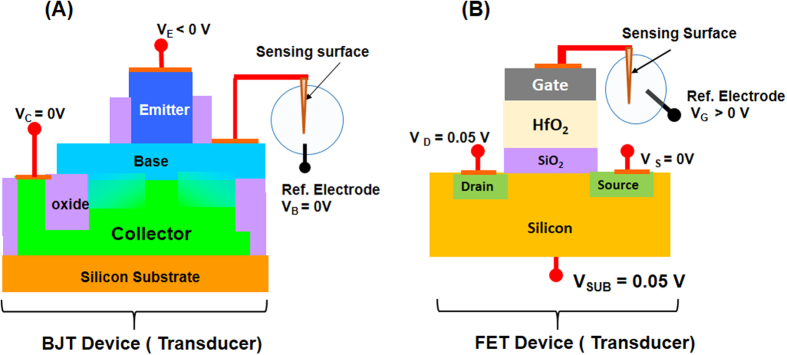
Electrochemical sensors with BJT and FET as transducers. Identical electrochemical sensors except for the transducer device type. (**A**) Schematic of an electrochemical sensor with a BJT device as the transducer; BJT base is connected to the silver chloride sensing surface that is immersed in the solution; a reference electrode is also immersed in the solution. Sensing signal (I_C_) is measured with the collector voltage (V_C_) and the base voltage V_B_ held at 0 V, whilst the emitter voltage (V_E_) is either varied or held constant. (**B**) Schematic of an electrochemical sensor with a FET device as the transducer; FET gate is connected to the silver chloride sensing surface that is immersed in the solution; a reference electrode is also immersed in the solution. Sensing signal (I_D_) is measured with drain voltage V_D_ = 50 mV, source (V_S_) and substrate (V_SUB_) voltages held at 0 V, whilst gate voltage V_G_ is either varied or held constant.

Figure 2(A) shows the schematic of an electrochemical sensor with bipolar junction transistor (BJT) as the transducer. The sensor consists of a NPN BJT device with its base connected to a silver chloride coated silver (AgCl/Ag) wire that forms the chloride sensitive sensing surface, in contact with the aqueous solution. A miniaturized reference electrode is also immersed in the solution. The BJT device is fabricated using standard silicon processing technology as discussed elsewhere^22^. Post fabrication, the BJT base is connected to an AgCl/Ag wire; the AgCl/Ag preparation is described in the Methods section. The BJT emitter area is 2.5 × 10^−7^ cm^2^ (25 μm^2^) and the sensing surface area is ~0.04 cm^2^. The voltages applied at the emitter, collector and reference electrode are V_E_, V_C_ and V_B_, respectively. The collector current (I_C_) is the sensing signal and sensing measurements are made with V_C_ = V_B_ = 0 V and V_E_ is either varied or set at a fixed value.

The equation for the sensing signal I_C_ is briefly reviewed^18^:


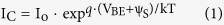



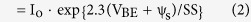


where, T is the temperature in Kelvin, *k* is the Boltzmann constant, I_o_ is a BJT device constant, and *q* is the electronic charge and V_BE_ = (V_B_ − V_E_). SS is the sub-threshold swing that is defined as the change in V_BE_ corresponding to a decade change in I_C_, therefore, SS = 2.3 kT/*q* from equation 1. In contrast, the sensing surface potential ψ_s_ depends solely on the sensing surface charge density associated with bound analyte, and is therefore a characteristic of the sensing surface/analyte interaction.

Figure 2(B) shows the schematic of an electrochemical sensor with a FET device as the transducer. The sensor has an N-type FET device with its gate connected to AgCl/Ag wire with AgCl forming the sensing surface in contact with the solution. A reference electrode is also immersed in the solution. The sensing surface is attached to the gate post FET fabrication. The silver chloride sensing surface, reference electrode and FET device area (=25 μm^2^) are the same as those for the BJT sensor. The FET device consists of a dual gate SiO_2_/HfO_2_ gate dielectric layer with TiN as the gate. SiO_2_ thickness is 1.5 nm and is grown by thermal oxidation at 900 °C, and HfO_2_ physical thickness is 2.0 nm and is deposited by atomic layer deposition (ALD) at 400 °C. The equivalent oxide thickness (EOT) of the dual layer stack is ~1.8 nm as determined by the accumulation capacitance. TiN thickness is 50 nm and is sputter deposited at 300 °C. More fabrication details of the HfO_2_ FET are given elsewhere^23^,^24^. These SiO_2_/HfO_2_ FETs are advantageous for the transducer application due their significantly lower gate currents in comparison to SiO_2_ gate dielectric FETs with heavily doped poly silicon gate. The optimized SiO_2_/HfO_2_ FET has a low gate current density of about 10^−3^/cm^2^ at a gate voltage of 1.0 V (Supplementary, Fig. S1). This current density is about 500 times lower than that in a standard SiO_2_ FET of similar gate dielectric thickness^25^. Since electrochemically induced sensing surface degradation depends on the gate current density, reduced gate current results in improved sensing surface life time. The threshold voltage, sub-threshold swing s(SS) and drain current hysteresis (ΔH) are other parameters impacting sensor performance. For example, low threshold voltage implies that the use voltage would be small, a low SS value results in enhanced sensitivity as discussed later, and small ΔH indicates that the sensing signal is reversible with gate voltage sweep. The optimized SiO_2_/HfO_2_ FET device has threshold voltage of ~0.24 V, SS = 71 mV/decade and ΔH < 1 mV (Supplementary, Fig. S1); these values are comparable to those for a SiO_2_ FET device. Hence, these SiO_2_/HfO_2_ FETs have significantly lower gate currents, an important advantage over SiO_2_ FETs.

The FET electrochemical sensor measurements are made as follows. The drain current (I_D_) is the sensing signal. The voltages applied at the drain, source, substrate and reference electrode are V_D_, V_S_, V_SUB_ and V_G_, respectively. Sensing measurements are made at room temperature with V_S_ = V_SUB_ = 0 V, V_D_ = 50 mV while V_G_ applied at the reference electrode is either varied or set at a fixed value. For an electrochemical sensor with FET transducer, the equation for the sensing current I_D_ is the same as the transfer curve equation for a standard FET device except V_G_ is replaced with (V_G_ + ψ_s_)^1^.

### Comparison between BJT and FET Transducers

**Figure 3:**
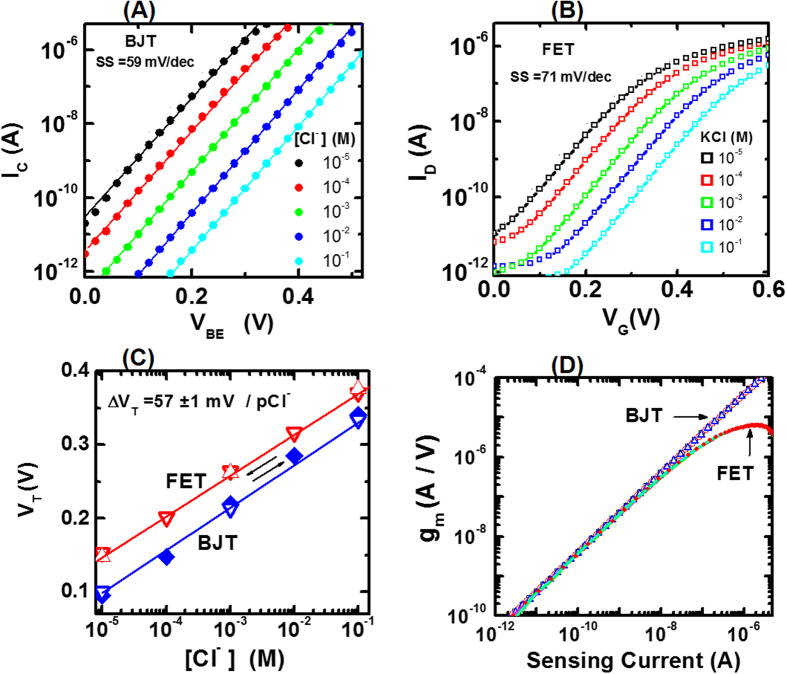
Sensing measurements using BJT and FET electrochemical sensors. (**A**) Dependence of the sensing signal I_C_ on applied voltage V_BE_ (=V_B_ − V_E_) in KCl solution of various concentrations for a BJT sensor; symbols are measurements and solid lines are fits. (**B**) Dependence of the sensing signal I_D_ on applied gate voltage V_G_ in KCl solution of various concentrations for a FET sensor; symbols are measurements and solid lines are fits. (**C**) Threshold voltage V_T_ dependence on chloride concentration for BJT and FET sensors; V_T_ values are extracted from transfer curves shown in (**A**) and (**B**); different symbols denote different V_T_ measurement sets; open symbols indicate that measurements are made as [Cl^−^] is increased, and filled symbols indicate that measurements are made as [Cl^−^] is decreased; arrows indicate that V_T_ dependence on chloride concentration is reversible; solid lines are fits and provide estimations of V_T_ shifts (ΔV_T_) with variation in chloride concentration by a decade: ΔV_T_ = 57 mV/pCl^−^ for both FET and BJT sensors. (**D**) Transconductance (g_m_) dependence on sensing current for BJT and FET sensors; I_C_ is the sensing current for the BJT sensor and I_D_ is the sensing current for the FET sensor; g_m_ values are extracted by numerically differentiating transfer curves shown in A and B.

Using the electrochemical sensors shown in Fig. 2, sensing measurements are performed for FET and BJT sensors. Measurements are performed in an aqueous KCl solution of varying concentrations (0.01–100 mM). Since KCl is fully ionized in water at room temperature, Cl^−^ and KCl concentrations are assumed to be the same. Figure 3A shows the dependence of the sensing signal I_C_ on the applied voltage V_BE_ measured at various chloride concentrations ([Cl^−^]) for the BJT sensor. Symbols denote measurements and solid lines are exponential fits to the data in accordance with equation 1. At each Cl^−^ concentration, I_C_ increases exponentially as V_BE_ increases with a sub-threshold swing (SS) of 59 mV/decade, consistent with the room temperature Nernst value. This SS value is same as that for the stand-alone BJT device (Supplementary, Fig. S2), and therefore SS is an intrinsic transducer property. Figure 3B shows the dependence of I_D_ on gate voltage (V_G_) measured at various [Cl^−^] for the FET sensor. At each Cl^−^ concentration, the sensing signal I_D_ increases exponentially with SS = 71 mV/decade at lower V_G_ and has a linear dependence at higher V_G_, consistent with the FET transfer curve equation. This SS = 71 mV/decade for the FET transducer is same as that for the stand-alone FET device (Supplementary, Fig. S1), thereby indicating once again SS is an intrinsic transducer property. From Fig. 3A,B three main observations can be made. (i) Sensing current versus applied voltage curves are significantly different for BJT and FET sensors. (ii) Sub-threshold swing are independent of chloride concentration for both sensors and SS for BJT sensor is smaller than that for the FET sensor. (iii) For both sensors, I_C_ and I_D_ curves shift as chloride concentration varies, thus indicating that threshold voltage V_T_ depends on [Cl^−^]; V_T_ is defined as the applied voltage value corresponding to a constant sensing current of 1 nA. As chloride concentration increases in the solution, more Cl^−^ bind to the sensing surface, thereby causing surface potential ψ_s_ to shift with a concomitant shift in V_T_: i.e. ΔV_T_ = Δψ_s_. Figure 3C shows dependence of V_T_ on [Cl^−^] for BJT and FET sensors: V_T_ varies reversibly with ΔV_T_ = 57 ± 1 mV/pCl for both sensors. This similarity is understandable since both electrochemical sensors have the same sensing surface with similar Δψ_s_ response. In summary, Fig. 3C shows that ΔV_T_ is independent of the transducer device choice.

Transconductance (g_m_) is another important property impacting sensor performance. g_m_ is defined as (δI_C_/δV_BE_) and (δI_D_/δV_G_) for BJT and FET sensors, respectively. Using data from Fig. 3A,B, g_m_ is estimated. Figure 3D shows the dependence of g_m_ on the sensing current for BJT and FET sensors. For sensing currents < 10 nA, g_m_ increases with a power law dependence on the sensing signal with an exponent of 0.99 at for both sensors, irrespective of the chloride concentration. At sensing currents > 10 nA, g_m_ corresponding to the FET sensor starts to decrease and is less than that for the BJT sensor. These g_m_ curves for BJT and FET transducers are similar to those observed for the stand-alone devices (Supplementary section, Fig. S3), thereby indicating that g_m_ is an intrinsic transducer property. Using these results from Fig. 3, the performance metrics for BJT and FET sensors are compared as discussed.

An electrochemical sensor sensitivity can be defined as the relative change in the sensing current (I) for a fixed change in the target analyte concentration. From equation 1, sensor sensitivity can be written as:


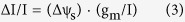


**Figure 4:**
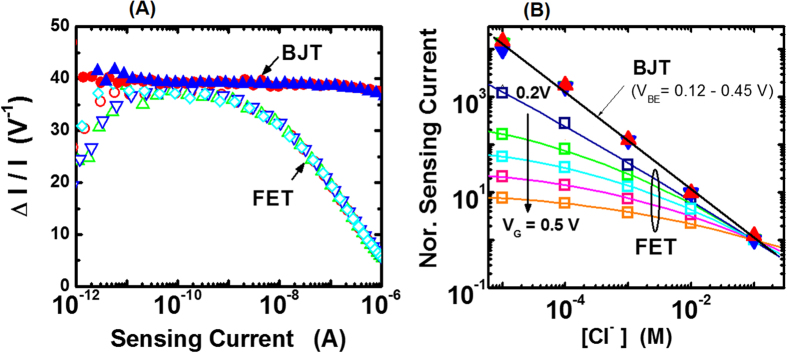
Sensitivity and calibration curves comparison for BJT and FET sensors. (**A**) BJT sensor sensitivity (ΔI_C_/I_C_) comparison with FET sensor sensitivity (ΔI_D_/I_D_); different colored symbols denote measurements made at different chloride concentrations; filled and open symbols correspond to BJT and FET sensors, respectively. (**B**) Comparison between normalized calibration curves for BJT and FET sensors; normalization is done by dividing the measured sensing current at different [Cl^−^] by the sensing current measured at 100 mM KCl solution; each curve is measured at a fixed applied voltage V_BE_ or V_G_ values as indicated by different colored symbols; solid lines are fits to data; normalized calibration curves for the BJT sensor are independent of the applied voltage V_BE_ value, whereas FET curves depend on the applied voltage V_G_.

In the above equation, Δψ_s_ is the change in the surface potential corresponding to a fixed analyte concentration change. Figure 3 shows that Δψ_s_ is same for both BJT and FET sensors. Hence, for the purpose of transducer comparison, Δψ_s_ can be set to 1 V and sensor sensitivities are estimated from equation 3. For the BJT sensor, sensitivity ΔI_C_/I_C_ = g_m_/I_C,_ where the transconductance is g_m_ = (δI_C_/δV_BE_). Similarly, the FET sensor sensitivity can be written as: ΔI_D_/I_D_ = (δI_D_/δV_G_)/I_D_. Using the transfer and transconductance curves shown in Fig. 3, sensor sensitivities at various chloride ion concentrations are estimated and compared. Figure 4A compares the BJT sensor sensitivity with the FET sensor sensitivity. The BJT sensor sensitivity (ΔI_C_/I_C_) is observed to be independent of I_C_, implying that sensing measurements can be made with the same high sensitivity over the entire I_C_ range of several decades, irrespective of applied V_BE_. In contrast, the FET sensor sensitivity ΔI_D_/I_D_ varies with the sensing signal, and therefore applied V_G_ has to be chosen such that I_D_ is in the range of 0.05 to 0. 5 nA to achieve maximum sensor sensitivity. Also, BJT sensor sensitivity (ΔI_C_/I_C_) is increasingly higher than the FET sensor sensitivity (ΔI_D_/I_D_) for sensing currents >1 nA. Hence, an electrochemical sensor with a BJT transducer is demonstrated to have superior sensitivity characteristics.

We now discuss calibration curves for BJT and FET electrochemical sensors: the calibration curve is defined as the sensing signal dependence on analyte concentration measured at a fixed applied voltage. To compare these two calibration curves, the sensing signal is normalized by the signal measured at 100 mM KCl concentration. Figure 4B shows the dependence of normalized sensing current on the chloride concentration for BJT and FET sensors at various applied voltages. For the BJT sensor, the normalized I_C_ increases by a factor of 10 per pCl, irrespective of the applied voltage V_BE_ value. In contrast, normalized calibration curve depends on the applied gate voltage V_G_ for the FET sensor. Normalized I_D_ increases by a factor of 8 per pCl over a narrow range of V_G_ ~ 0.2 to 0.25 V and this dependence becomes weaker at higher V_G_. In summary, normalized calibration curves are independent of applied voltage for a BJT sensor, and therefore it is better suited for mobile sensing applications.

Signal to noise ratio (SNR) is another important sensor performance metric. As discussed elsewhere^26^, SNR measures the sensor resolution (i.e. smallest measurable change in ion concentration) and can be written as:


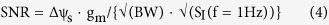


where, S_I_ (f = 1 Hz) is the sensing current noise power density at 1 Hz, g_m_ is the transconductance, and band width BW = ln (f_2_/f_1_) for low frequency cutoff f_1_ and high frequency cutoff f_2_ in the measurement bandwidth. Using equation 4 and assuming BW = 1 and Δψ_s_ = 1.0 V, the SNR per volt can be written as^26^:


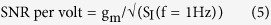


**Figure 5:**
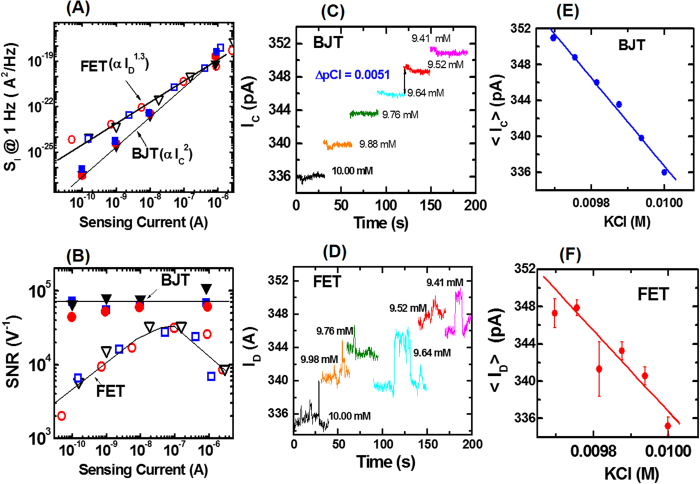
Sensing current noise power density (S_1_), signal noise ratio (SNR) and signal resolution comparison: (**A**) Dependence of S_I_ at 1 Hz on sensing current for BJT and FET sensors; I_C_ and I_D_ are sensing currents for BJT and FET sensors, respectively; blue symbols denote S_1_ measured in 1 mM KCl, red symbols denote S_1_ measured in 100 mM KCL, and black symbols denote measurements made on a standard device with no sensing surface or solution; solid lines are power law fits and extracted exponent values are shown in the figure. (**B**) Comparison of SNR dependence on sensing current for BJT and FET sensors; blue symbols denote measurements made in 1 mM KCl, red symbols denote measurements made in 100 mM KCL, and black symbols denote measurements made on a standard device with no sensing surface or solution. (**C**) BJT sensor signal I_C_ dependence as 10 mM KCl solution of 1.6 mL volume is diluted by adding 20 μL of deionized water at each step; I_C_ is measured at a fixed V_BE_ = 0.260 V. (**D**) FET sensor signal I_D_ dependence on as 10 mM KCl solution of 1.6 mL volume is diluted by adding 20 μL of deionized water at each step; I_D_ is measured at a fixed V_G_ = 0.284 V. (**E**) Mean and standard deviation (error bar) values of sensing signal I_C_ at various dilution steps; symbols denote mean I_C_ (<I_C_>) estimated from BJT sensor data shown in (**C**); solid line is a power law fit. (**F**) Mean and standard deviation values of sensing signal I_D_ at various dilution steps; symbols denote mean I_D_ (<I_D_>) estimated from FET sensor data shown in (**D**); solid line is a power law fit.

Using equation 5, the SNR is estimated as follows. Sensing current noise power density at 1 Hz (S_I_) and transconductance (g_m_) are measured for BJT and FET sensors in KCl solution of varying concentrations, and also for the stand alone device (i.e. no sensing surface or solution). To measure S_I_ for the BJT sensor, the collector current is measured as a function of time at a sampling rate of 50 ms at different V_BE_ values with V_C_ = V_B_ = 0 V. The collector current time domain data is analyzed using the Fast Fourier Transform Welch method^27^ and the I_C_ noise power density (S_I_) as a function of frequency is estimated. Similar noise measurements associated with I_D_ are performed for the FET sensor. Figure 5A shows the dependence of S_I_ on the sensing current for sensors with BJT and FET transducers. For both sensors, S_I_ shows no dependence on the chloride concentration and is the same as for the stand-alone device without the sensing surface and solution. This implies that the measured sensor noise is mainly due the transducer. S_1_ has a power law dependence on the sensing current with an exponent of 2 and 1.3 for BJT and FET sensors, respectively. Also, S_1_ is lower for the BJT sensor, particularly at sensing currents <10^−7^ A. Transconductance (g_m_) values for BJT and FET sensors are estimated from Fig. 3D. Using the noise data (Fig. 5A) and g_m_ (Fig. 3D), signal to noise ratio (SNR) values are calculated from equation 5 for the two sensors. Figure 5B compares the signal to noise ratio (SNR) for the two chloride ion sensors. The SNR is observed to be independent of the chloride concentration and is the same as for the stand-alone device (i.e. no solution or sensing surface) for both sensors, implying that the transducer device is the dominant cause of the measured noise. For the BJT sensor, the SNR is higher and remains constant over the entire sensing range. This is a significant advantage because the signal can be measured with the same high SNR, irrespective of applied voltage V_BE_. In contrast, the maximum SNR is observed at I_D_ ~100 nA for the FET sensor, and therefore the corresponding V_G_ has to be carefully chosen to achieve maximum SNR. From Figs 4A and 5B, we observe that the maximum sensitivity and SNR occur at different sensing current values, and as a result a tradeoff has to be made between sensitivity and SNR for a FET sensor. Since SNR is a measure of the sensor resolution, the above SNR observations are also true for the sensor resolution.

To further highlight the sensor resolution dependence on the transducer choice, a dilution experiment is performed using BJT and FET sensors. Starting with a 10 mM KCl solution of 1.6 mL volume, the solution is diluted in steps by adding 20 μL of deionized water. Each dilution step results in chloride concentration change of ΔpCl ~0.0051. To ensure that the comparison is accurate, the applied voltage (V_BE_ or V_G_) is fixed at a value such that the corresponding sensing current at the initial 10 mM KCl concentration is the same. At each dilution step, the sensing current is measured for about 100 sec after a wait of about 1 minute. Figure 5C shows the BJT sensing signal I_C_ measured at a fixed V_BE_ = 0.26 V at various dilution steps. Figure 5D shows dilution experiment results for a FET sensor, where the sensing signal I_D_ is measured at a fixed V_G_ = 0.284 V. Figures 4C and 5D measurements are analyzed and results are shown in Fig. 5E,F. Figure 5E shows mean and standard deviation values of BJT sensing current I_C_ at various dilution steps. Mean I_C_ value increases by ΔI_C_ ~3 pA per dilution step and the standard deviation δI_C_ ~ ±0.15 pA. Since the ratio ΔI_C_/δI_C_ ~ 10 and ΔpCl ~ 0.0051 at each dilution step, estimated BJT sensor resolution is about 0.0005 which is the highest reported value to the best of our knowledge^20^,^21^. Figure 5F shows mean and standard deviation values of sensing current I_D_ at various dilution steps for the FET sensor. Mean I_D_ value increases by ΔI_C_ ~ 2.2 pA per dilution step and the standard deviation δI_C_ ~ ± 1 pA. Since signal change and signal noise are of comparable magnitude for the FET sensor, FET sensor is unable to resolve ΔpCl ~ 0.0051 at each dilution step. A key observation from Fig. 5C,D is that sensing signal I_D_ has higher (about 6 times) noise in comparison to that for I_C_, thus indicating that the FET transducer is noisier than the BJT transducer. This higher noise in I_D_ can be attributed to the presence of gate dielectric related Si/SiO_2_ interfacial traps in the FET device, whereas the BJT device has no such gate dielectric related interfacial traps. In summary, the BJT sensor has lower noise and therefore has higher resolution in comparison to the FET sensor.

Another difference between these two electrochemical sensors is the voltage applied to the aqueous solution by the reference electrode. In the case of a BJT sensor, the reference electrode applies 0 V to the solution during sensing measurements. In contrast for the FET sensor, the reference electrode applies a voltage |V_G_ | > 0 V to the solution during sensing measurements. This |V_G_ | > 0 V can cause interference, particularly if the sensing is being performed on voltage gated ion channel proteins such as those embedded in neuron membranes. Hence, BJT sensors are better suited for a wider range of biological systems. Lastly, like a FET transducer, a BJT is also compatible with silicon processing technology and can be integrated with automation circuitry on a single chip. Both FET and BJT can easily be miniaturized to 0.1 um^2^ but the FET is easier to further scale down. In summary, this comparative study demonstrates that the electrochemical sensor with a BJT as the transducer has significantly enhanced sensing characteristics in comparison to that with a FET transducer.

### Cl^−^ Sensing in Artificial Human Sweat for Diagnostic Applications

**Figure 6:**
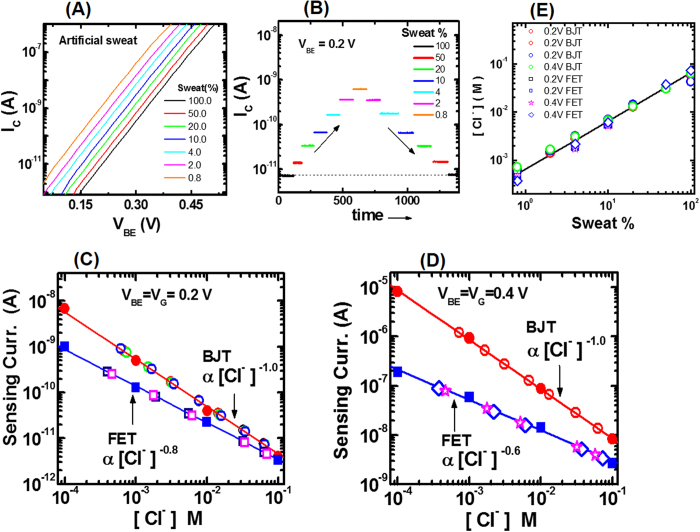
Chloride sensing measurements in artificial human sweat. (**A**) Dependence of BJT sensing current I_C_ on applied voltage V_BE_ for diluted artificial human sweat samples with sweat percentages varying from 100% to 0.8%. (**B**) Reversibility and repeatability of the BJT sensing signal I_C_ for samples with various sweat percentages. (**C**) Estimation of chloride concentration in samples with varying sweat percentages from sensing currents measured V_BE_ = V_G_ = 0.2 V for BJT and FET sensors; solid lines denote calibration curves obtained from power law fits to KCl solution data (filled symbols), and open symbols denote measurements in sweat samples. (**D**) Estimation of chloride concentrations in samples with sweat percentages from I_C_ and I_D_ measured at V_BE_ = V_G_ = 0.4 V for BJT and FET sensors; solid lines denote calibration curves obtained from power law fits to KCl solution data (filled symbols), and open symbols denote measurements in sweat samples; exponents for power law fits corresponding to data for BJT and FET sensors are shown in the figure. (**E**) Comparison between measured and calculated chloride concentrations in samples with sweat percentages varying from 100% to 0.8%.; solid line is the calculated curve and open symbols are chloride concentrations estimated from (**C**) and (**D**).

Since electrochemical sensors with BJT as transducers have significantly enhanced sensing characteristics, they are further investigated for as a potential candidate for hand-held diagnostic application for Cystic Fibrosis disease that requires the measurement of [Cl^−^] in human eccrine sweat^19^. Chloride sensing measurements are performed using artificial human sweat that are similar in chemical composition to the human eccrine sweat. Measurements are performed on a set of seven artificial sweat samples that have been diluted by varying amounts with ultrapure water: the sweat percentage ranged from 100 (no dilution) to 0.8 (125 times dilution). Figure 6A shows measured BJT sensor transfer curves for undiluted and diluted sweat samples. For each sample, I_C_ increases with increasing V_BE_ in accordance with equation 2 with an exponent that corresponds to 25 °C. This dependence is similar to that observed in aqueous KCl solutions (Fig. 3A). Also, I_C_ curves are observed to shift towards the left with decreasing sweat percentages, thereby indicating that [Cl^−^] is decreasing. In Fig. 6B, the sensing signal (I_C_) reversibility and reproducibility in sweat is investigated. I_C_ is measured for about 100 sec at a fixed V_BE_ = 0.2 V for each artificial sweat sample. Measurements are made on samples in the order of first decreasing and then increasing sweat concentration. I_C_ is observed to be repeatable and reversible within 4% of error. Though, the focus is on BJT sensor, similar sensing measurements are also performed with the FET sensor to verify that chloride sensitivity comparisons are same in sweat as those of Fig. 4B. In Fig. 6C, the solid line denotes the chloride calibration curves for BJT and FET sensors, obtained by fitting sensing signals measured in for KCl solutions (solid symbols) at constant applied voltage V_BE_ = V_G_ = 0.2 V. From the fits, the calibration curve equation for the BJT sensor is I_C_ = 4.3 × 10^−13^[Cl^−^]^−1.03^, and the calibration curve equation for the FET sensor is I_D_ = 5.5 × 10^−13^[Cl^−^]^−0.80^. Using calibration curves and sensing signals at V_BE_ = V_G_ = 0.2 V (open symbols), chloride concentration values are estimated for undiluted and diluted sweat samples for both BJT and FET sensors. Similar analysis is also performed for BJT and FET sensing currents measured at another applied voltage V_BE_ = V_G_ = 0.4 V as shown in Fig. 6D. From Fig. 6C,D, two main observations can be made. (i) Sensing signal increases with a power law dependence on the [Cl^−^] with an exponent value of −1.0 that is independent of the applied voltage V_BE_ for the BJT sensor, whereas the exponent decreases from −0.8 V to −0.6 as the applied voltage V_G_ increases from 0.2 V to 0.4 V. (ii) Since the exponent magnitude is higher for the BJT sensor data, the BJT sensor has higher chloride sensitivity than the FET sensor. These two observations obtained using sweat samples are consistent with those obtained using KCl solutions as shown in Fig. 4B. To verify the accuracy of sensor measurements, chloride concentrations are also calculated by using the supplier provided chloride concentration value of 65 mM for the undiluted sweat and the known sweat dilution percentage. The calculated and measured chloride concentration results are compared in Fig. 6E; solid line denote calculated results and open symbols are [Cl^−^] measurements from Fig. 6C,D using BJT and FET sensors. Measurements are in agreement with calculated values of chloride levels in artificial sweat samples for both sensors.

**Figure 7:**
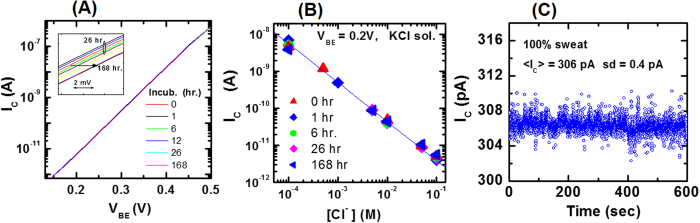
Sensing measurements in artificial sweat using BJT sensor. (**A**) Measured I_C_ dependence on applied V_BE_ as a function of incubation time in 100% artificial human sweat at room temperature; the inset shows that curves shifts by ~3 mV after a week of incubation. (**B**) Measured calibration curve as a function of incubation time. (**C**) Repeated (6000 times) measurement of the signal I_C_ at V_BE_ = 0.3 V in undiluted artificial sweat; mean I_C_ = 306 pA with standard deviation (sd) of 0.4 pA.

Since artificial sweat has amino acids, minerals and metabolites, it is possible that these amino acids and metabolites would nonspecifically bind to the sensing surface, and thus cause signal drifts or degrade the sensitivity to chloride ions. To evaluate these effects, the silver chloride sensing surface is incubated in 100% artificial sweat for total time of 168 hours (1 week); intermittently the incubation is interrupted and chloride sensing measurements are made in the incubating sweat. Figure 7A shows the measured dependence of I_C_ on V_BE_ after various incubation times. From the figure, the transfer curves show a small shift of ~3 mV after a week of incubation. Since the curve shifted back when re-measured in fresh sweat, the observed drift is attributed to a slight increase in the chloride ion concentration due to the sweat evaporation during the long incubation period. Hence, the sensing signal shows negligible (<1 mV) drift after one week of incubation in artificial human sweat. This result is understandable because sweat is mostly (99%) water with low concentrations of biomolecules and as a result non-specific binding is minimal.

To measure the impact of week long incubation in sweat on the sensing surface sensitivity to chloride ions, the calibration curve at a fixed V_BE_ = 0.2 V is measured as a function of incubation time as shown in Fig. 7B. The calibration curve (I_C_ versus [Cl^−^]) remains unchanged with incubation time, thus indicating that the silver chloride sensing surface is not degraded with prolonged incubation in sweat. At the end of the week long incubation, the sensing signal I_C_ at a fixed V_BE_ = 0.3 V is repeatedly measured 6000 times in sweat as shown in Fig. 7C. The signal shows no drifts with repeated measurements and has 0.13% noise which is similar to the noise measured before incubation. In summary, incubation in artificial sweat does not induce signal drifts, calibration curve degradation or noise increase for the BJT sensor.

## Conclusion

BJT and FET transducers are compared by performing sensing measurements on two electrochemical sensors that are identical in all details, except for the transducer device type. This comparative study demonstrates that an electrochemical sensor with the BJT transducer has significantly enhanced sensing characteristics in comparison to that with the widely used FET transducer. The BJT sensor has sensitivity and resolution and calibration curves that are independent of the use voltages. Hence, BJT sensors are particularly well suited for making high sensitivity and resolution sensing measurements with minimal calibration requirements and are well suited for mobile sensing applications. As a demonstration for mobile diagnostic applications, the BJT electrochemical sensor is investigated by measuring chloride levels in artificial human sweat and is shown to be a viable option for portable cystic fibrosis diagnosis.

## Methods

### Chloride ion sensing surface preparation

The silver chloride is used as the chloride ion (Cl^−^) sensing surface for both BJT and FET sensors. It is prepared by the electrochemical anodization of a silver (Ag) wire (Sigma Aldrich, part# 265586, ≥99.99%; 1 mm diameter) in a chloride solution. To prepare a silver chloride coated silver wire, a silver wire is first cleaned by sonication in ethanol (~5 mins) followed by rinse in ultrapure Millipore water. The cleaned silver wire is used as the anode and a platinum wire (Sigma Aldrich, part# 267201, 99.99%) is used as the cathode in an electrolytic cell. Since KCl^28^, HCl^29^ and their mixtures^30^ have been widely used as an electrolyte for anodization of the Ag wire, we have evaluated different electrolytic solution with the aim of identifying the best recipe. The results are summarized in Table S1 in Supporting Information Section. As shown in Table S1, the recipe that uses 1 M HCl as the electrolytic solution with a constant current of 2 mA/cm^2^ for 15 minutes produced a silver chloride surface with highest chloride sensitivity and least drifts during electrical measurements. The silver chloride surface prepared by this recipe is used for all sensing measurements.

### Materials used in sensing measurements

All sensing measurements are performed in air at room temperature using either potassium chloride (99.5%, Sigma Aldrich) aqueous solution using or synthetic human Eccrine sweat (Pickering Laboratories, http://www.pickeringtestsolutions.com) of 100 μL volume. Ultrapure water with resistivity of 18.2 MΩ at 298 K is used for making potassium chloride (KCl) solutions and for sweat dilution. The reference electrode is a leak free commercially available reference electrode (Innovative Instruments, Inc., Florida) with outer diameter of 1 mm.

## Additional Information

**How to cite this article**: Zafar, S. *et al*. Comparison between Field Effect Transistors and Bipolar Junction Transistors as Transducers in Electrochemical Sensors. *Sci. Rep.* **7**, 41430; doi: 10.1038/srep41430 (2017).

**Publisher's note:** Springer Nature remains neutral with regard to jurisdictional claims in published maps and institutional affiliations.

## Supplementary Material

Supplementary Dataset 1

## Figures and Tables

**Figure 1:**
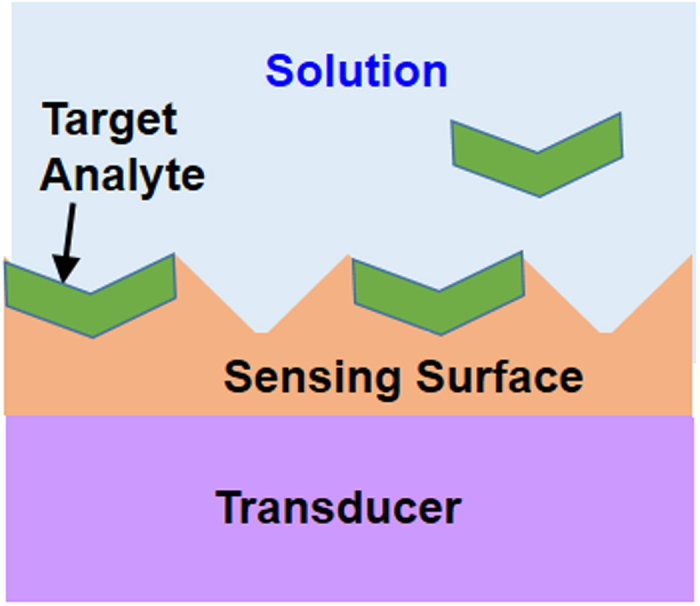
An electrochemical sensor schematic. Sensor components are shown: sensing surface and transducer; the sensing surface is in contact with the solution with dissolved target analyte that selectively bind to the sensing surface.
