# Supplementary material for: Comparison between Field Effect Transistors and Bipolar Junction Transistors as Transducers in Electrochemical Sensors
Source: Sci Rep. 2017 Jan 30;7:41430. doi: 10.1038/srep41430 (PMC5278393; doi:10.1038/srep41430)
Supplement: Supplementary Dataset 1 [file srep41430-s1.doc]

**Supplementary Information for Manuscript titled (SREP-16-36795):**

**“**Comparison between Field Effect Transistors and Bipolar Junction Transistors as Transducers in Electrochemical Sensors**”, S**ufi Zafar, Minhua Lu and Ashish Jagtiani

| **Solution** | **Current**  **Density**  **(mA/cm^2^)** | **Time (min)** | **Mean Drift Rate**  **(mV/min)** | | |
| --- | --- | --- | --- | --- | --- |
|  |  |  | **10mM** | **50mM** | **100mM** |
| 1M KCl | 0.2 | 25 | 0.040 | 0.160 | 0.180 |
| 1M KCl | 0.5 | 10 | 0.140 | 0.400 | 0.320 |
| 1M KCl | 0.96 | 5 | 0.20 | 0.260 | 0.040 |
| 1M KCl | 0.026 | 90 | 0.14 | 0.320 | 0.360 |
| 1M KCl+ 1M HCl | 0.2 | 25 | 0.03 | 0.100 | 0.040 |
| 1M HCl | 2 | 15 | < 0.01 | < 0.01 | < 0.01 |
| 1M KCl + 0.25M HCl | 0.23 | 25 | 0.010 | 0.040 | 380 |

**Table S1**: Silver chloride (AgCl) sensing surface preparation recipes.

A silver chloride (AgCl) film is deposited on the surface of the silver (Ag) wire by applying a DC voltage across the two electrodes. A constant current was maintained across the two electrodes. Table 1 shows the different electrolytic solutions, current densities and times used to prepare AgCl/Ag electrodes. Once the AgCl surface is prepared, it is rinsed in deionized water and tested for electrical stability and sensitivity. Stability of each AgCl/Ag electrode is tested by measuring potential drift against a commercial reference electrode (Accumet, part#13-620-53) in 10 mM, 50 mM and 100 mM KCl solution over first 5 minutes of immersion into the solution. As shown in Table 1, the AgCl surface corresponding to 1 M of HCl electrolytic solution recipe shows the best result: lowest mean drift rates in the first 5 minutes of immersion in the solution and are observed to reach steady state in about a minute. Also, the chloride ion sensitivity is 57.1 mV/pCl (~Nernst limit) for the AgCl prepared by this 1 M HCl recipe. In summary, the AgCl sensing surface used in the entire study was prepared by the optimized recipe with 1M HCl electrolytic solution.

**
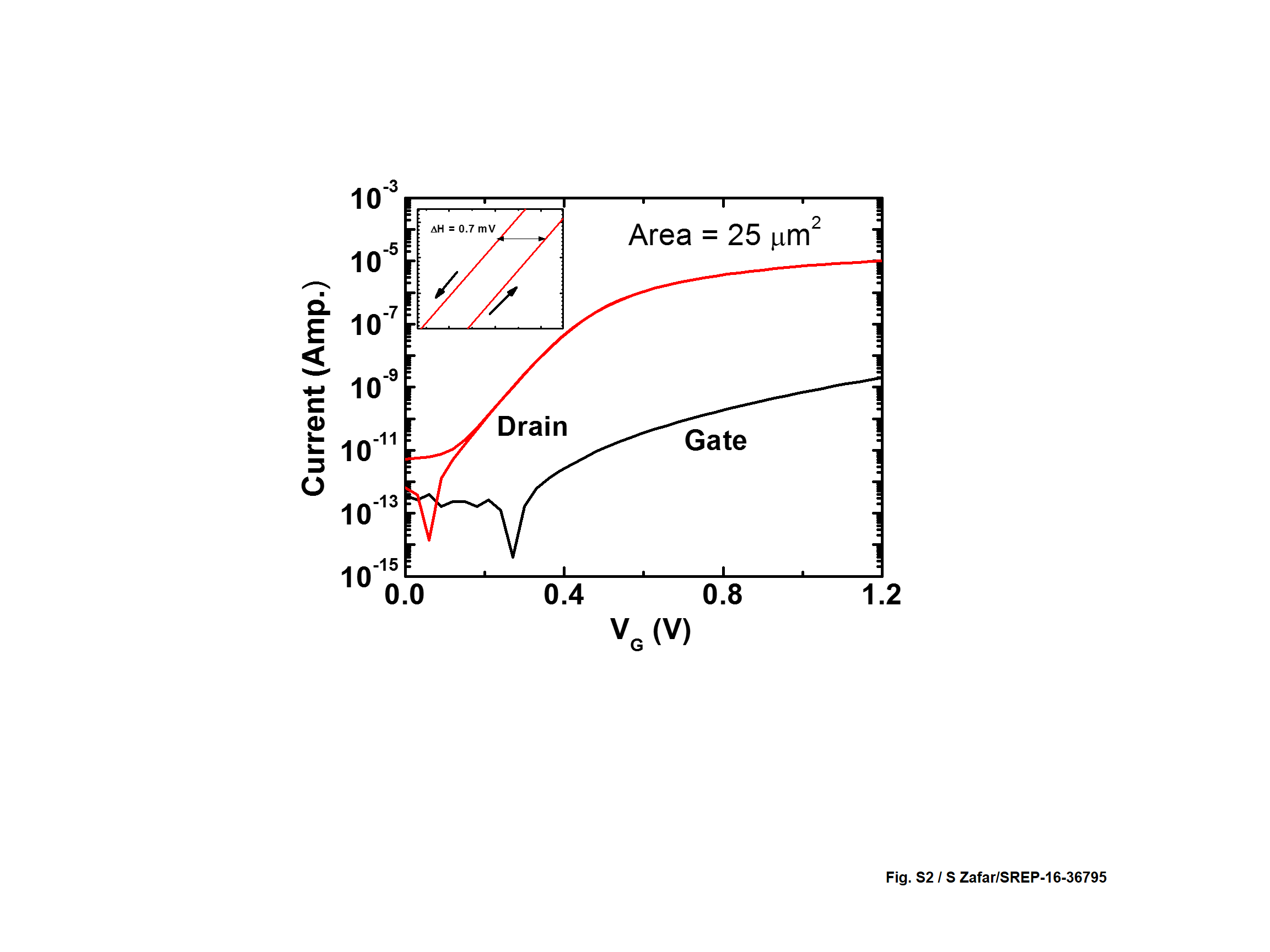
**

**Figure S1: Transfer Curve for SiO_2_/HfO_2_ FET device.** Dependence of drain current I_D_ (red solid line) and gate current I_G_ (black solid line) on the applied gate voltage V_G_ for the stand alone FET (no sensing surface or solution); sub-threshold swing (SS) =71 mV/decade and V_T_ = 0.24 V; inset shows that the drain current has < 1 mV of hysteresis during double voltage sweep.


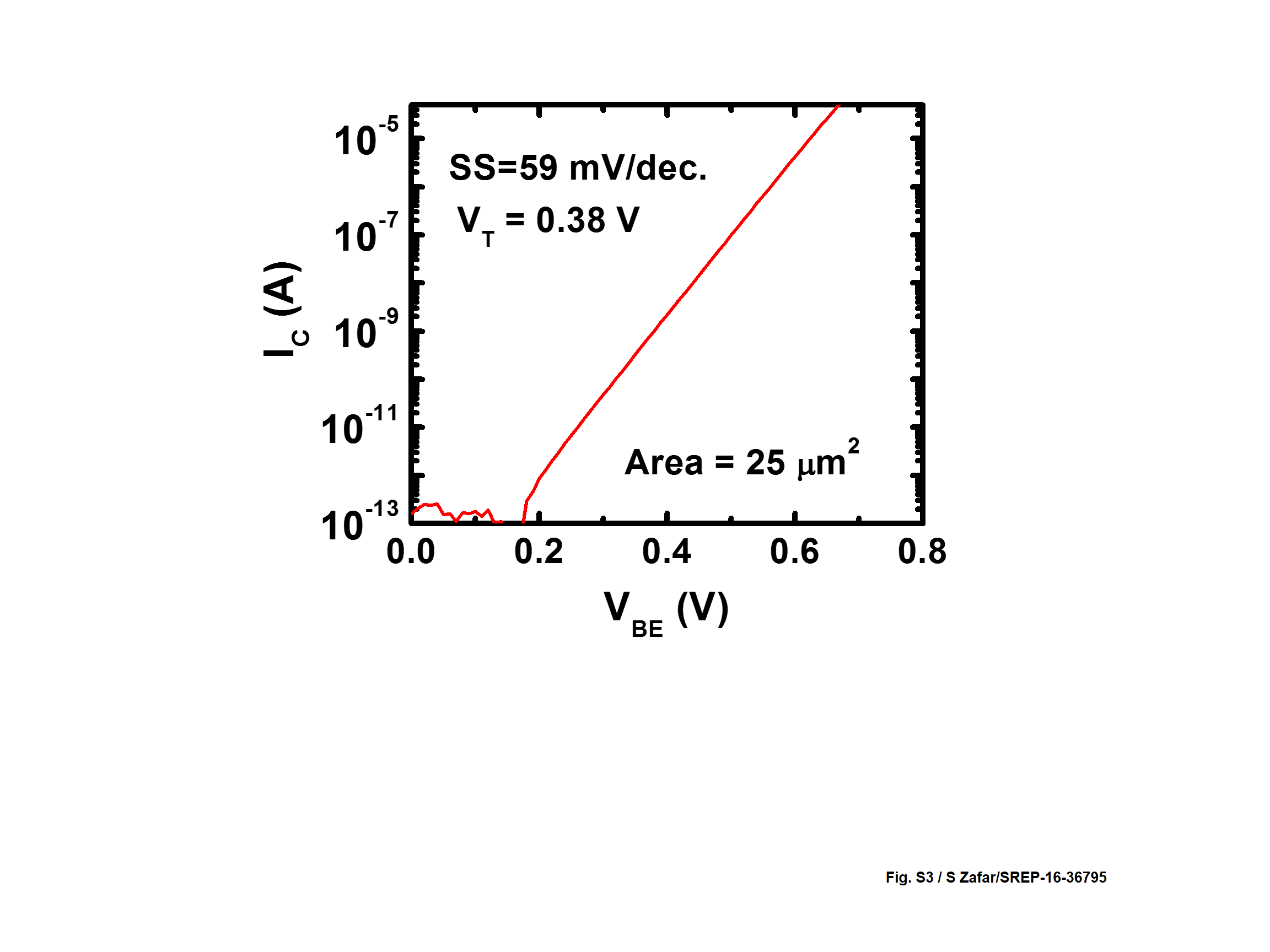


**Figure S2: Transfer Curve for the BJT device:** Dependence of collector current on the applied voltage V_BE_; SS is the sub-threshold swing and the threshold V_T_ = is defined as the V_BE_ value corresponding to I_C_ = 1 nA.


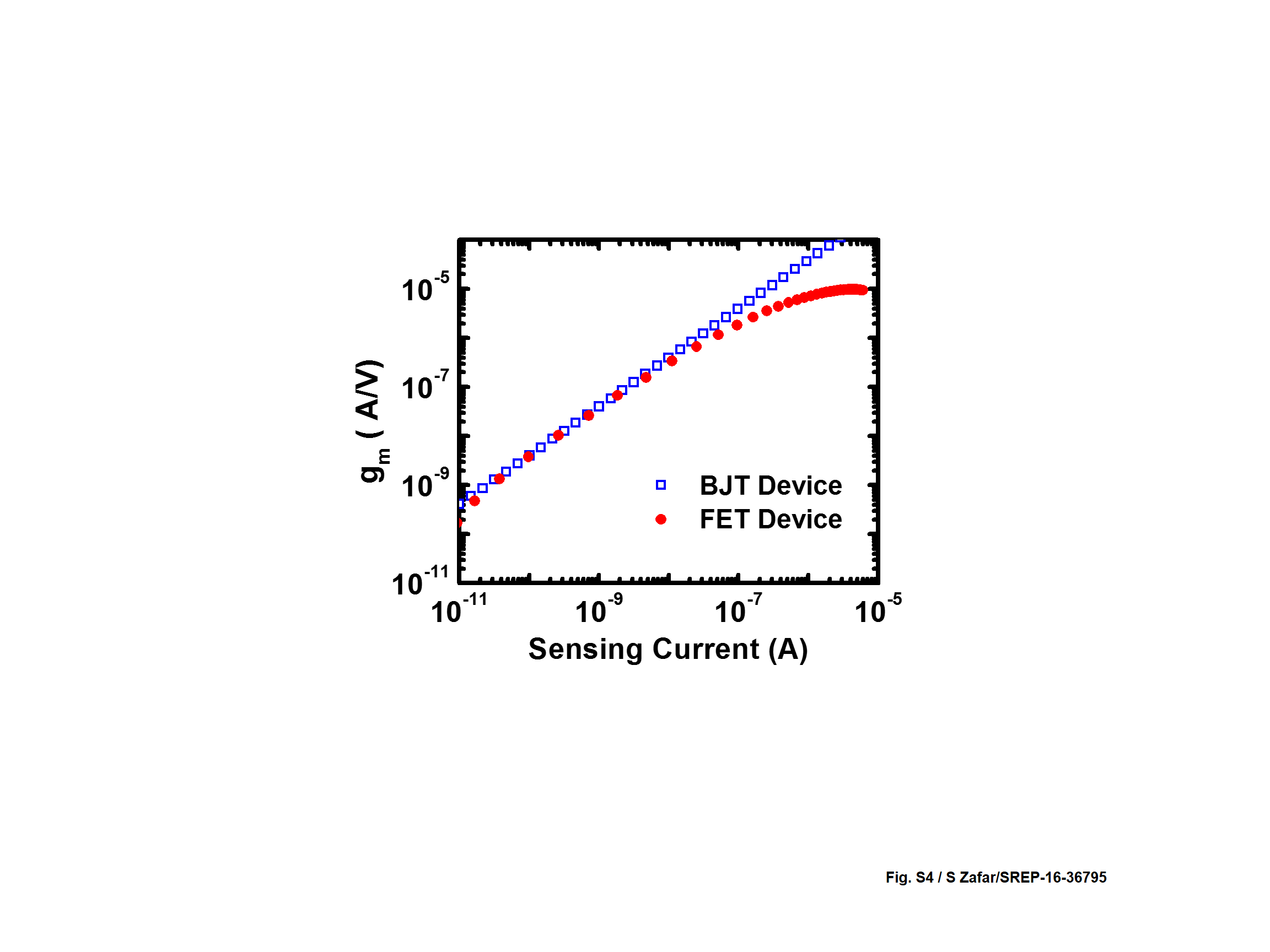


**Figure S3: Transconductance (g_m_) curves for BJT and FET devices:** Dependence of g_m_ on the sensing signal for BJT and FET stand–alone devices (no sensing surface and solution).

on
